# *N*-[(4-Chloro­phen­yl)sulfon­yl]acetamide

**DOI:** 10.1107/S1600536812033764

**Published:** 2012-08-04

**Authors:** Hoong-Kun Fun, Tze Shyang Chia, K. Jyothi, Poornima Hegde, Pramila Rita D’Souza

**Affiliations:** a X-ray Crystallography Unit, School of Physics, Universiti Sains Malaysia, 11800 USM, Penang, Malaysia; b Department of Chemistry, St. Joseph Engineering College, Vamanjoor, Mangalore 575 028, Karnataka, India

## Abstract

The asymmetric unit of the title compound, C_8_H_8_ClNO_3_S, consists of two crystallographically independent mol­ecules (*A* and *B*). The dihedral angles between the benzene ring and amide C—C(=O)—NH– plane are 87.6 (3) (mol­ecule *A*) and 86.0 (3)° (mol­ecule *B*). In the crystal, the independent mol­ecules are alternately linked by N—H⋯O hydrogen bonds into an infinite chain along the *b* axis. Short inter­molecular Cl⋯Cl contacts [3.2882 (5) and 3.2812 (5) Å] are also observed.

## Related literature

For a related structure, see: Fun *et al.* (2012 ▶). For the stability of the temperature controller used in the data collection, see: Cosier & Glazer (1986 ▶).
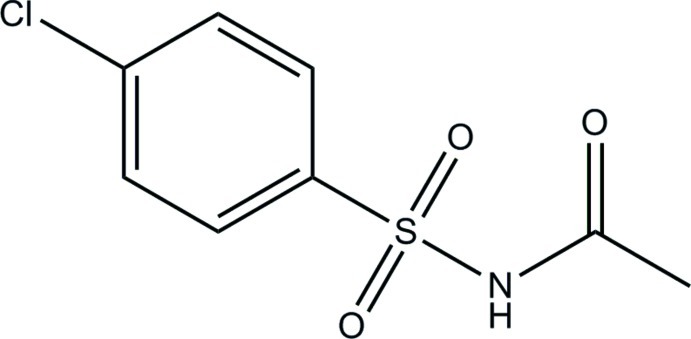


## Experimental

### 

#### Crystal data

C_8_H_8_ClNO_3_S
*M* *_r_* = 233.66
Monoclinic, 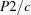

*a* = 12.1801 (6) Å
*b* = 9.2529 (4) Å
*c* = 17.6769 (8) Å
β = 101.979 (1)°
*V* = 1948.83 (16) Å^3^
*Z* = 8
Mo *K*α radiation
μ = 0.59 mm^−1^
*T* = 100 K
0.36 × 0.14 × 0.14 mm

#### Data collection

Bruker APEX DUO CCD area-detector diffractometer
Absorption correction: multi-scan (*SADABS*; Bruker, 2009 ▶) *T*_min_ = 0.819, *T*_max_ = 0.923
45479 measured reflections
7130 independent reflections
5439 reflections with *I* > 2σ(*I*)
*R*_int_ = 0.033

#### Refinement

*R*[*F* ^2^ > 2σ(*F* ^2^)] = 0.028
*wR*(*F* ^2^) = 0.090
*S* = 1.04
7130 reflections
263 parameters
H atoms treated by a mixture of independent and constrained refinement
Δρ_max_ = 0.48 e Å^−3^
Δρ_min_ = −0.52 e Å^−3^

### 

Data collection: *APEX2* (Bruker, 2009 ▶); cell refinement: *SAINT* (Bruker, 2009 ▶); data reduction: *SAINT*; program(s) used to solve structure: *SHELXTL* (Sheldrick, 2008 ▶); program(s) used to refine structure: *SHELXTL*; molecular graphics: *SHELXTL*; software used to prepare material for publication: *SHELXTL* and *PLATON* (Spek, 2009 ▶).

## Supplementary Material

Crystal structure: contains datablock(s) global, I. DOI: 10.1107/S1600536812033764/is5173sup1.cif

Structure factors: contains datablock(s) I. DOI: 10.1107/S1600536812033764/is5173Isup2.hkl

Supplementary material file. DOI: 10.1107/S1600536812033764/is5173Isup3.cml

Additional supplementary materials:  crystallographic information; 3D view; checkCIF report

## Figures and Tables

**Table 1:** Hydrogen-bond geometry (Å, °)

*D*—H⋯*A*	*D*—H	H⋯*A*	*D*⋯*A*	*D*—H⋯*A*
N1*B*—H1*NB*⋯O3*A* ^i^	0.871 (15)	1.939 (15)	2.7980 (10)	168.6 (13)
N1*A*—H1*NA*⋯O3*B* ^ii^	0.865 (15)	1.939 (15)	2.7952 (10)	170.0 (13)

Symmetry codes: (i) 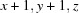; (ii) 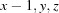.

**Fig. 1.**
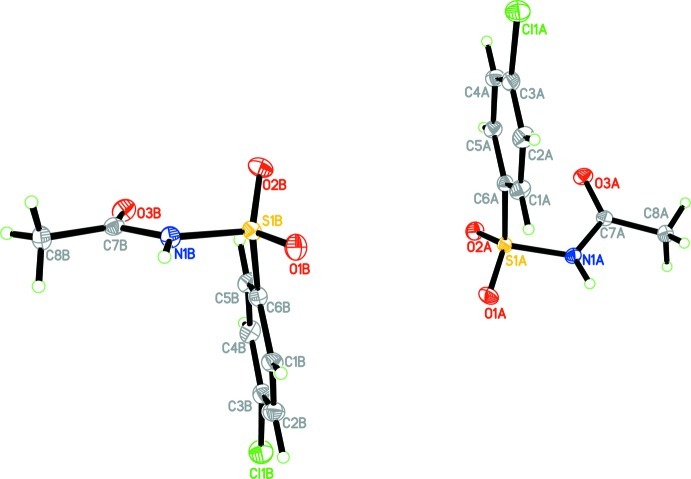
The molecular structure of the title compound with atom labels and 50% probability displacement ellipsoids.

**Fig. 2.**
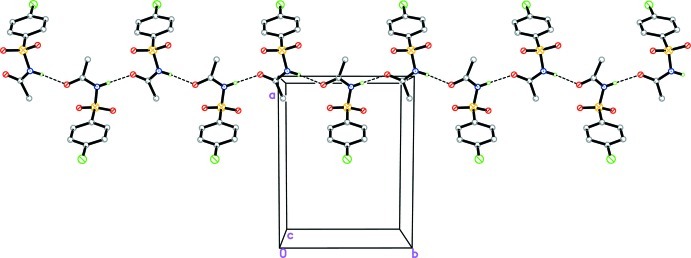
The crystal packing of the title compound viewed along the c axis. The dashed lines represent the hydrogen bonds. For clarity sake, hydrogen atoms not involved in hydrogen bonding have been omitted.

## supplementary crystallographic information

### Comment

In continuation to our reports on the biological activity of sulfonamide containing compounds (Fun *et al.*, 2012), we report herein the crystal structure of the title compound.

The asymmetric unit of the title compound (Fig. 1), consists of two crystallographically independent molecules (*A* and *B*). The C═ O and N—H bonds in the amide planes [C7A/O3A/N1A/H1NA and C7B/O3B/N1B/H1NB; maximum deviations = 0.043 (5) Å at atom N1A and 0.047 (5) Å at atom H1NB] are *trans* to each other. The benzene ring (C1–C6) forms a dihedral angle of 87.6 (3)° with the amide plane in molecule *A*, whereas the corresponding angle is 86.0 (3)° in molecule *B*. The bond lengths and angles are comparable to those found in a related structure (Fun *et al.*, 2012). In the crystal (Fig. 2), molecules are linked by intermolecular N1B—H1NB···O3A and N1A—H1NA···O3B hydrogen bonds (Table 1) into an infinite chain along the *b* axis. Short intermolecular Cl1A···Cl1A [3.2882 (5) Å; 1 - *x*, 1 - *y*, 1 - *z*] and Cl1B···Cl1B [3.2812 (5) Å; 1 - *x*, *y*, -1/2 - *z*] are also observed.

### Experimental

To a vigorously stirred mixture of 4-chlorobenzenesulphonamide and silica sulfuric acid, acid chloride or acid anhydride was added at RT. The progress of the reaction was monitored by TLC. After completion of the reaction, ethyl acetate was added and the solid catalyst was removed by filtration. The filtrate was washed with water, dried and evaporated. The crude product was purified by recrystallization from an ethanol solution to yield colourless single crystals of the title compound.

### Refinement

The N-bound H atoms were located in a difference Fourier map and refined freely [N1A—H1NA = 0.865 (14) Å and N1B—H1NB = 0.871 (14) Å]. The remaining H atoms were positioned geometrically (C—H = 0.95 and 0.98 Å) and refined using a riding model with *U*_iso_(H) = 1.2 or 1.5*U*_eq_(C). A rotating group model was applied to the methyl group. Four outliers, (204), (100), (348) and (233), were omitted in the final refinement.

### Crystal data

**Table:**

C_8_H_8_ClNO_3_S	*F*(000) = 960
*M**_r_* = 233.66	*D*_x_ = 1.593 Mg m^−^^3^
Monoclinic, *P*2/*c*	Mo *K*α radiation, λ = 0.71073 Å
Hall symbol: -P 2yc	Cell parameters from 9840 reflections
*a* = 12.1801 (6) Å	θ = 2.5–32.6°
*b* = 9.2529 (4) Å	µ = 0.59 mm^−^^1^
*c* = 17.6769 (8) Å	*T* = 100 K
β = 101.979 (1)°	Block, colourless
*V* = 1948.83 (16) Å^3^	0.36 × 0.14 × 0.14 mm
*Z* = 8	

### Data collection

**Table:**

Bruker APEX DUO CCD area-detector diffractometer	7130 independent reflections
Radiation source: fine-focus sealed tube	5439 reflections with *I* > 2σ(*I*)
Graphite monochromator	*R*_int_ = 0.033
φ and ω scans	θ_max_ = 32.7°, θ_min_ = 2.2°
Absorption correction: multi-scan (*SADABS*; Bruker, 2009)	*h* = −18→18
*T*_min_ = 0.819, *T*_max_ = 0.923	*k* = −14→13
45479 measured reflections	*l* = −26→26

### Refinement

**Table:**

Refinement on *F*^2^	Primary atom site location: structure-invariant direct methods
Least-squares matrix: full	Secondary atom site location: difference Fourier map
*R*[*F*^2^ > 2σ(*F*^2^)] = 0.028	Hydrogen site location: inferred from neighbouring sites
*wR*(*F*^2^) = 0.090	H atoms treated by a mixture of independent and constrained refinement
*S* = 1.04	*w* = 1/[σ^2^(*F*_o_^2^) + (0.0436*P*)^2^ + 0.5704*P*] where *P* = (*F*_o_^2^ + 2*F*_c_^2^)/3
7130 reflections	(Δ/σ)_max_ = 0.001
263 parameters	Δρ_max_ = 0.48 e Å^−^^3^
0 restraints	Δρ_min_ = −0.52 e Å^−^^3^

### Special details

**Table:**

Experimental. The crystal was placed in the cold stream of an Oxford Cryosystems Cobra open-flow nitrogen cryostat (Cosier & Glazer, 1986) operating at 100.0 (1) K.
Geometry. All e.s.d.'s (except the e.s.d. in the dihedral angle between two l.s. planes) are estimated using the full covariance matrix. The cell e.s.d.'s are taken into account individually in the estimation of e.s.d.'s in distances, angles and torsion angles; correlations between e.s.d.'s in cell parameters are only used when they are defined by crystal symmetry. An approximate (isotropic) treatment of cell e.s.d.'s is used for estimating e.s.d.'s involving l.s. planes.
Refinement. Refinement of *F*^2^ against ALL reflections. The weighted *R*-factor *wR* and goodness of fit *S* are based on *F*^2^, conventional *R*-factors *R* are based on *F*, with *F* set to zero for negative *F*^2^. The threshold expression of *F*^2^ > σ(*F*^2^) is used only for calculating *R*-factors(gt) *etc*. and is not relevant to the choice of reflections for refinement. *R*-factors based on *F*^2^ are statistically about twice as large as those based on *F*, and *R*- factors based on ALL data will be even larger.

### Fractional atomic coordinates and isotropic or equivalent isotropic displacement parameters (Å^2^)

**Table:**

	*x*	*y*	*z*	*U*_iso_*/*U*_eq_	
Cl1A	0.47973 (2)	0.51064 (3)	0.405269 (18)	0.02673 (7)	
S1A	0.170782 (19)	0.51977 (2)	0.076741 (14)	0.01367 (6)	
O1A	0.19060 (6)	0.64897 (8)	0.03653 (4)	0.02048 (14)	
O2A	0.17384 (6)	0.38233 (8)	0.04030 (4)	0.01910 (14)	
O3A	0.03482 (6)	0.33069 (7)	0.15483 (4)	0.01845 (14)	
N1A	0.04580 (7)	0.54746 (8)	0.09688 (5)	0.01463 (14)	
C1A	0.29561 (8)	0.64713 (10)	0.20474 (6)	0.01909 (18)	
H1AA	0.2712	0.7362	0.1802	0.023*	
C2A	0.36445 (8)	0.64567 (11)	0.27788 (6)	0.02079 (19)	
H2AA	0.3879	0.7335	0.3042	0.025*	
C3A	0.39837 (8)	0.51313 (11)	0.31194 (6)	0.01803 (19)	
C4A	0.36704 (8)	0.38278 (11)	0.27505 (6)	0.01842 (18)	
H4AA	0.3926	0.2939	0.2994	0.022*	
C5A	0.29771 (7)	0.38417 (10)	0.20193 (6)	0.01646 (17)	
H5AA	0.2745	0.2962	0.1757	0.020*	
C6A	0.26276 (8)	0.51635 (9)	0.16772 (6)	0.01437 (17)	
C7A	−0.00697 (7)	0.44855 (9)	0.13606 (5)	0.01418 (16)	
C8A	−0.11629 (9)	0.49932 (10)	0.15322 (7)	0.01856 (19)	
H8AA	−0.1411	0.4314	0.1888	0.028*	
H8AB	−0.1729	0.5044	0.1050	0.028*	
H8AC	−0.1062	0.5953	0.1771	0.028*	
Cl1B	0.52074 (2)	0.97797 (3)	−0.155284 (17)	0.02637 (7)	
S1B	0.82941 (2)	1.00993 (2)	0.172976 (15)	0.01367 (6)	
O1B	0.80290 (6)	1.13770 (8)	0.21146 (4)	0.01954 (14)	
O2B	0.83407 (6)	0.87333 (8)	0.21118 (4)	0.01972 (14)	
O3B	0.97057 (6)	0.83316 (7)	0.09436 (4)	0.01887 (14)	
N1B	0.95264 (7)	1.04795 (8)	0.15212 (5)	0.01459 (14)	
C1B	0.69388 (8)	1.12533 (10)	0.04604 (6)	0.01691 (17)	
H1BA	0.7112	1.2157	0.0712	0.020*	
C2B	0.62534 (8)	1.11904 (10)	−0.02700 (6)	0.01873 (18)	
H2BA	0.5950	1.2048	−0.0526	0.022*	
C3B	0.60174 (8)	0.98501 (11)	−0.06205 (6)	0.01785 (18)	
C4B	0.64282 (8)	0.85698 (11)	−0.02594 (6)	0.01874 (18)	
H4BA	0.6241	0.7667	−0.0508	0.022*	
C5B	0.71197 (8)	0.86331 (10)	0.04738 (6)	0.01664 (17)	
H5BA	0.7415	0.7773	0.0732	0.020*	
C6B	0.73726 (8)	0.99763 (9)	0.08230 (6)	0.01408 (17)	
C7B	1.00893 (7)	0.95272 (9)	0.11338 (5)	0.01431 (16)	
C8B	1.11652 (8)	1.00994 (10)	0.09647 (7)	0.01848 (19)	
H8BA	1.1475	0.9396	0.0651	0.028*	
H8BB	1.1704	1.0264	0.1452	0.028*	
H8BC	1.1019	1.1012	0.0681	0.028*	
H1NB	0.9739 (11)	1.1378 (16)	0.1584 (8)	0.025 (3)*	
H1NA	0.0217 (11)	0.6354 (16)	0.0901 (8)	0.026 (3)*	

### Atomic displacement parameters (Å^2^)

**Table:**

	*U*^11^	*U*^22^	*U*^33^	*U*^12^	*U*^13^	*U*^23^
Cl1A	0.02335 (13)	0.03545 (14)	0.01876 (15)	−0.00052 (9)	−0.00167 (10)	−0.00159 (9)
S1A	0.01605 (11)	0.01243 (10)	0.01325 (12)	0.00092 (7)	0.00468 (8)	0.00067 (7)
O1A	0.0248 (3)	0.0179 (3)	0.0203 (4)	−0.0010 (3)	0.0084 (3)	0.0060 (3)
O2A	0.0240 (3)	0.0163 (3)	0.0169 (3)	0.0032 (2)	0.0043 (3)	−0.0037 (2)
O3A	0.0202 (3)	0.0117 (3)	0.0232 (4)	−0.0001 (2)	0.0038 (3)	0.0033 (2)
N1A	0.0165 (3)	0.0101 (3)	0.0179 (4)	0.0015 (2)	0.0050 (3)	0.0020 (3)
C1A	0.0213 (4)	0.0135 (4)	0.0215 (5)	0.0011 (3)	0.0024 (4)	−0.0019 (3)
C2A	0.0220 (4)	0.0175 (4)	0.0219 (5)	0.0002 (3)	0.0022 (4)	−0.0048 (3)
C3A	0.0146 (4)	0.0225 (4)	0.0165 (5)	−0.0007 (3)	0.0024 (4)	−0.0009 (3)
C4A	0.0175 (4)	0.0177 (4)	0.0196 (5)	−0.0004 (3)	0.0027 (3)	0.0028 (3)
C5A	0.0166 (4)	0.0135 (4)	0.0190 (4)	−0.0004 (3)	0.0029 (3)	0.0010 (3)
C6A	0.0148 (4)	0.0132 (4)	0.0154 (5)	0.0002 (3)	0.0040 (3)	−0.0007 (3)
C7A	0.0156 (4)	0.0129 (4)	0.0137 (4)	−0.0015 (3)	0.0023 (3)	0.0001 (3)
C8A	0.0176 (4)	0.0199 (4)	0.0196 (5)	0.0018 (3)	0.0071 (4)	0.0023 (3)
Cl1B	0.02278 (12)	0.03587 (14)	0.01794 (14)	−0.00114 (9)	−0.00155 (10)	−0.00122 (9)
S1B	0.01612 (11)	0.01213 (9)	0.01342 (12)	−0.00106 (7)	0.00458 (9)	−0.00113 (7)
O1B	0.0222 (3)	0.0182 (3)	0.0193 (4)	0.0006 (2)	0.0067 (3)	−0.0061 (3)
O2B	0.0251 (3)	0.0159 (3)	0.0181 (3)	−0.0030 (2)	0.0044 (3)	0.0039 (2)
O3B	0.0216 (3)	0.0118 (3)	0.0227 (4)	0.0011 (2)	0.0033 (3)	−0.0030 (2)
N1B	0.0169 (3)	0.0099 (3)	0.0179 (4)	−0.0010 (2)	0.0055 (3)	−0.0013 (3)
C1B	0.0198 (4)	0.0125 (4)	0.0187 (4)	−0.0007 (3)	0.0047 (3)	0.0005 (3)
C2B	0.0192 (4)	0.0173 (4)	0.0193 (5)	0.0000 (3)	0.0032 (3)	0.0028 (3)
C3B	0.0143 (4)	0.0227 (4)	0.0162 (5)	−0.0008 (3)	0.0024 (4)	−0.0006 (3)
C4B	0.0174 (4)	0.0177 (4)	0.0203 (5)	−0.0007 (3)	0.0019 (3)	−0.0051 (3)
C5B	0.0167 (4)	0.0127 (4)	0.0200 (5)	0.0001 (3)	0.0027 (3)	−0.0022 (3)
C6B	0.0148 (4)	0.0125 (4)	0.0157 (5)	−0.0008 (3)	0.0048 (4)	−0.0010 (3)
C7B	0.0160 (4)	0.0126 (4)	0.0140 (4)	0.0022 (3)	0.0022 (3)	0.0004 (3)
C8B	0.0172 (4)	0.0192 (4)	0.0204 (5)	−0.0004 (3)	0.0071 (4)	−0.0006 (3)

### Geometric parameters (Å, º)

**Table:**

Cl1A—C3A	1.7394 (11)	Cl1B—C3B	1.7375 (11)
S1A—O2A	1.4295 (7)	S1B—O2B	1.4286 (7)
S1A—O1A	1.4366 (7)	S1B—O1B	1.4339 (7)
S1A—N1A	1.6537 (8)	S1B—N1B	1.6559 (8)
S1A—C6A	1.7591 (11)	S1B—C6B	1.7593 (11)
O3A—C7A	1.2200 (11)	O3B—C7B	1.2206 (11)
N1A—C7A	1.3839 (11)	N1B—C7B	1.3823 (11)
N1A—H1NA	0.865 (14)	N1B—H1NB	0.871 (14)
C1A—C2A	1.3873 (15)	C1B—C2B	1.3855 (14)
C1A—C6A	1.3943 (13)	C1B—C6B	1.3949 (13)
C1A—H1AA	0.9500	C1B—H1BA	0.9500
C2A—C3A	1.3907 (14)	C2B—C3B	1.3895 (14)
C2A—H2AA	0.9500	C2B—H2BA	0.9500
C3A—C4A	1.3864 (14)	C3B—C4B	1.3887 (14)
C4A—C5A	1.3897 (14)	C4B—C5B	1.3925 (14)
C4A—H4AA	0.9500	C4B—H4BA	0.9500
C5A—C6A	1.3913 (13)	C5B—C6B	1.3934 (12)
C5A—H5AA	0.9500	C5B—H5BA	0.9500
C7A—C8A	1.5012 (13)	C7B—C8B	1.4998 (13)
C8A—H8AA	0.9800	C8B—H8BA	0.9800
C8A—H8AB	0.9800	C8B—H8BB	0.9800
C8A—H8AC	0.9800	C8B—H8BC	0.9800
			
O2A—S1A—O1A	119.64 (5)	O2B—S1B—O1B	119.73 (5)
O2A—S1A—N1A	110.25 (4)	O2B—S1B—N1B	110.14 (4)
O1A—S1A—N1A	103.55 (4)	O1B—S1B—N1B	103.54 (4)
O2A—S1A—C6A	108.95 (4)	O2B—S1B—C6B	109.19 (4)
O1A—S1A—C6A	109.08 (4)	O1B—S1B—C6B	108.77 (4)
N1A—S1A—C6A	104.24 (4)	N1B—S1B—C6B	104.31 (4)
C7A—N1A—S1A	123.31 (6)	C7B—N1B—S1B	122.73 (6)
C7A—N1A—H1NA	120.8 (9)	C7B—N1B—H1NB	120.4 (9)
S1A—N1A—H1NA	114.7 (9)	S1B—N1B—H1NB	115.7 (9)
C2A—C1A—C6A	119.21 (9)	C2B—C1B—C6B	119.35 (9)
C2A—C1A—H1AA	120.4	C2B—C1B—H1BA	120.3
C6A—C1A—H1AA	120.4	C6B—C1B—H1BA	120.3
C1A—C2A—C3A	118.68 (9)	C1B—C2B—C3B	118.81 (9)
C1A—C2A—H2AA	120.7	C1B—C2B—H2BA	120.6
C3A—C2A—H2AA	120.7	C3B—C2B—H2BA	120.6
C4A—C3A—C2A	122.38 (10)	C4B—C3B—C2B	122.34 (10)
C4A—C3A—Cl1A	118.79 (8)	C4B—C3B—Cl1B	118.92 (8)
C2A—C3A—Cl1A	118.81 (8)	C2B—C3B—Cl1B	118.73 (8)
C3A—C4A—C5A	118.96 (9)	C3B—C4B—C5B	118.85 (9)
C3A—C4A—H4AA	120.5	C3B—C4B—H4BA	120.6
C5A—C4A—H4AA	120.5	C5B—C4B—H4BA	120.6
C4A—C5A—C6A	118.98 (9)	C4B—C5B—C6B	119.03 (9)
C4A—C5A—H5AA	120.5	C4B—C5B—H5BA	120.5
C6A—C5A—H5AA	120.5	C6B—C5B—H5BA	120.5
C5A—C6A—C1A	121.79 (9)	C5B—C6B—C1B	121.61 (9)
C5A—C6A—S1A	119.51 (7)	C5B—C6B—S1B	120.13 (7)
C1A—C6A—S1A	118.67 (7)	C1B—C6B—S1B	118.23 (7)
O3A—C7A—N1A	121.09 (8)	O3B—C7B—N1B	120.88 (8)
O3A—C7A—C8A	124.21 (8)	O3B—C7B—C8B	124.43 (8)
N1A—C7A—C8A	114.69 (8)	N1B—C7B—C8B	114.68 (8)
C7A—C8A—H8AA	109.5	C7B—C8B—H8BA	109.5
C7A—C8A—H8AB	109.5	C7B—C8B—H8BB	109.5
H8AA—C8A—H8AB	109.5	H8BA—C8B—H8BB	109.5
C7A—C8A—H8AC	109.5	C7B—C8B—H8BC	109.5
H8AA—C8A—H8AC	109.5	H8BA—C8B—H8BC	109.5
H8AB—C8A—H8AC	109.5	H8BB—C8B—H8BC	109.5
			
O2A—S1A—N1A—C7A	50.89 (9)	O2B—S1B—N1B—C7B	51.56 (9)
O1A—S1A—N1A—C7A	−179.98 (8)	O1B—S1B—N1B—C7B	−179.27 (8)
C6A—S1A—N1A—C7A	−65.90 (8)	C6B—S1B—N1B—C7B	−65.50 (8)
C6A—C1A—C2A—C3A	−0.07 (15)	C6B—C1B—C2B—C3B	0.04 (14)
C1A—C2A—C3A—C4A	0.83 (16)	C1B—C2B—C3B—C4B	1.14 (16)
C1A—C2A—C3A—Cl1A	−177.29 (8)	C1B—C2B—C3B—Cl1B	−177.27 (7)
C2A—C3A—C4A—C5A	−1.11 (16)	C2B—C3B—C4B—C5B	−1.32 (16)
Cl1A—C3A—C4A—C5A	177.02 (7)	Cl1B—C3B—C4B—C5B	177.08 (8)
C3A—C4A—C5A—C6A	0.60 (15)	C3B—C4B—C5B—C6B	0.32 (15)
C4A—C5A—C6A—C1A	0.14 (15)	C4B—C5B—C6B—C1B	0.83 (15)
C4A—C5A—C6A—S1A	−177.64 (7)	C4B—C5B—C6B—S1B	−177.14 (7)
C2A—C1A—C6A—C5A	−0.41 (15)	C2B—C1B—C6B—C5B	−1.02 (15)
C2A—C1A—C6A—S1A	177.39 (8)	C2B—C1B—C6B—S1B	176.99 (7)
O2A—S1A—C6A—C5A	−18.56 (9)	O2B—S1B—C6B—C5B	−21.27 (9)
O1A—S1A—C6A—C5A	−150.79 (8)	O1B—S1B—C6B—C5B	−153.56 (8)
N1A—S1A—C6A—C5A	99.12 (8)	N1B—S1B—C6B—C5B	96.44 (8)
O2A—S1A—C6A—C1A	163.58 (8)	O2B—S1B—C6B—C1B	160.70 (8)
O1A—S1A—C6A—C1A	31.36 (9)	O1B—S1B—C6B—C1B	28.41 (9)
N1A—S1A—C6A—C1A	−78.73 (8)	N1B—S1B—C6B—C1B	−81.59 (8)
S1A—N1A—C7A—O3A	−3.57 (13)	S1B—N1B—C7B—O3B	−1.87 (13)
S1A—N1A—C7A—C8A	175.90 (7)	S1B—N1B—C7B—C8B	177.08 (7)

### Hydrogen-bond geometry (Å, º)

**Table:**

*D*—H···*A*	*D*—H	H···*A*	*D*···*A*	*D*—H···*A*
N1*B*—H1*NB*···O3*A*^i^	0.871 (15)	1.939 (15)	2.7980 (10)	168.6 (13)
N1*A*—H1*NA*···O3*B*^ii^	0.865 (15)	1.939 (15)	2.7952 (10)	170.0 (13)

Symmetry codes: (i) *x*+1, *y*+1, *z*; (ii) *x*−1, *y*, *z*.
